# ADVANCING RESEARCH ACROSS THE ADRD CONTINUUM IN UNDERREPRESENTED POPULATIONS

**DOI:** 10.1093/geroni/igad104.0357

**Published:** 2023-12-21

**Authors:** Laura Zahodne, Kristine Ajrouch, Cerise Elliott

## Abstract

This symposium highlights innovative work advancing ADRD research with African Americans, Latinos, and Arab Americans. Included papers address critical issues along the ADRD spectrum from life course risk factors, through mild cognitive impairment (MCI), to dementia caregiving. Investigating links between the early-life environment and later-life ADRD risk, Dr. Tsotsoros examines cognitive correlates of adverse childhood experiences (ACEs) in two disparate samples, demonstrating the need to characterize risk pathways among older Latina women, who are at increased risk of both ACEs and ADRD. Using nationally representative data, Dr. Esiaka focuses on adult risk factors for ADRD among Black men. She shows that neighborhood physical disorder and poor sleep quality represent key risk factors for worse cognitive health in this critically understudied population. Moving along the ADRD continuum, Dr. Darwish focuses on another severely underrepresented group: Arab American immigrants with MCI. Based on in-depth work with older adults in Lebanon, she details cultural adaptations made in the development of a combined cognitive-behavioral and cognitive rehabilitation intervention for this group. Next, Dr. Bouldin uses nationally representative data on African American, Latino, and non-Latino White older adults with dementia to quantify racial/ethnic differences in the prevalence and correlates of caregiver support service use. Findings highlight both risk and resilience among dementia caregivers from minoritized racial/ethnic groups. Finally, Cerise Elliott from the National Institute on Aging (NIA) will offer perspectives on how disaggregating population subgroups through between- and within-group research designs can advance NIA’s goal of understanding and eliminating racial/ethnic inequalities across the ADRD continuum. This is an Alzheimer’s Disease and Related Dementias Interest Group Sponsored Symposium.

